# Open Wedge High Tibial Osteotomy with Distal Tubercle Osteotomy Lessens Change in Patellar Position

**DOI:** 10.1155/2017/4636809

**Published:** 2017-07-18

**Authors:** Hoon Park, Hyun Woo Kim, Jin Hwa Kam, Dong Hoon Lee

**Affiliations:** ^1^Department of Orthopaedic Surgery, Gangnam Severance Hospital, Yonsei University College of Medicine, 211 Eonju-ro, Gangnam-gu, Seoul 06273, Republic of Korea; ^2^Division of Orthopaedic Surgery, Severance Children's Hospital, Yonsei University College of Medicine, 50 Yonsei-ro, Seodaemun-gu, Seoul 03722, Republic of Korea

## Abstract

The purpose of this study was to investigate the change in patellar position after open wedge high tibial osteotomy (OWHTO) with distal tubercle osteotomy (DTO), comparing outcomes of conventional OWHTO in young adults with proximal tibia varus deformity but no arthritic manifestations. Thirty-three patients (mean age, 31.8 years) subjected to OWHTO/DTO were matched with 30 patients (mean age, 33.5 years) undergoing conventional OWHTO. Patellar position, as measured in pre- and postoperative standing lateral radiographs, was compared. Patellar height was assessed via Insall-Salvati ratio, modified Insall-Salvati ratio, Blackburne-Peel (BP) index, Caton-Deschamps (CD) index, and modified Miura-Kawamura index. Computed tomography was used to measure lateral patellar tilt and shift. In the OWHTO group, all patellar height indices decreased significantly following surgery. Although mean values of BP and CD indices decreased significantly in the OWHTO/DTO group, other determinants of patellar height showed no significant postoperative differences. Significant postoperative declines in average lateral patellar tilt were also evident in both groups, but pre- and postoperative lateral patellar shift did not differ significantly. OWHTO/DTO can be performed without significant changes in patellar height. The results obtained support that OWHTO/DTO is suitable for relatively young patients with proximal tibia vara but no arthritic change.

## 1. Introduction

Genu varum deformity of the lower extremity results in abnormal load distribution across the knee joint and may lead to medial compartment joint osteoarthritis [1]. Although various methods of osteotomy have emerged to correct proximal tibial varus deformities, open wedge high tibial osteotomy (OWHTO) has gained popularity in recent years as the preferred approach in relatively young patients with varus malalignment and medial compartment knee osteoarthritis [2, 3]. Unfortunately, change in patellofemoral indices (i.e., patellar height, tilt, and shift) is a potential postoperative complication of OWHTO [4–7]. Such deviations, resulting from distalization of the tibial tuberosity [6, 8], may alter patellofemoral biomechanics to culminate in patellofemoral joint problems and difficulty in later total knee arthroplasty [9–12].

Technical modifications of OWHTO have been extensively studied in efforts to prevent change in patellar height. Whereas OWHTO undertaken below the tibial tuberosity is one option to mitigate the risk of patella infera, this technique has the disadvantage of cortical bone involvement and slow healing rate [13]. Alternatively, OWHTO with distal tubercle osteotomy (OWHTO/DTO) has been proposed to minimize any impact on patellar height [8]. Using this technique, which incorporates the tibial tubercle into the proximal plateau fragment, patellar position is less vulnerable. Previous studies have proven that, compared with OWHTO alone, OWHTO/DTO is effective in minimizing patellar height loss [8, 14, 15].

In active young adults with proximal tibial vara but no pain or arthritic change on medial side of knee, it is particularly important to minimize patellar position after correcting the deformity. To the best of our knowledge, however, there have been no studies evaluating lateral inclination and deviation in patellar indices after OWHTO using computed tomography (CT). Hence, the purpose of this study was to investigate the change in patellar position after corrective surgery, comparing outcomes of these two surgical techniques (OWHTO versus OWHTO/DTO). We anticipated that OWHTO/DTO would prove superior to OWHTO in this regard.

## 2. Materials and Methods

### 2.1. Study Patients

This study was a retrospective comparative investigation approved by the Institutional Review Board at our institution. A total of 155 consecutive patients who had undergone bilateral OWHTO or OWHTO/DTO for idiopathic proximal tibial vara between January 2011 and June 2015 were identified by electronic search of medical records. Patients with available preoperative CT images and follow-up CT images after index surgeries were included. At our institution, preoperative CT scans are done routinely as part of surgical planning, typically with follow-up CT imaging 6 months after surgery to evaluate locations and angles of osteotomies. Both preoperative and follow-up CT scans were performed as dictated by patient management protocols of referring surgeons and were not confined to symptomatic patients. Inclusion criteria were as follows: (1) skeletally mature patients younger than 40 years of age with cosmetic purpose; (2) idiopathic proximal tibia vara without pain or arthritic change; and (3) no history of fracture or ligamentous injury, congenital bony deformities, or infections of the lower extremity. Patients with patellofemoral joint symptoms, rheumatoid arthritis, or limited range of motion were excluded from the study.

Ultimately, 63 patients (126 tibias) were enrolled and stratified by surgical technique. All patients underwent bilateral surgery but only the right tibia of each patient was analyzed to limit the number of variables. A single surgeon performed all surgeries. The surgeon switched the surgical technique during the study period. Group 1 consisted of 30 patients (30 tibias) who underwent OWHTO between January 2011 and February 2013. Group 2 consisted of 33 patients (33 tibias) who were treated with OWHTO/DTO from March 2013 and June 2015. Preoperative data, including age, sex, and body mass index (BMI), were recorded in each instance.

### 2.2. Surgical Technique

Fixator-assisted high tibial osteotomy was performed by one senior surgeon [16]. All patients were operated on in supine position, using a conventional operating table and general anesthesia. Initially, a 2.5-cm oblique incision was made midway between the center of the tibial tuberosity and the posteromedial border of the proximal tibia, starting 1 cm below the upper margin of the pes anserinus tendon, then continuing proximally and in parallel with the posteromedial border of the tibia. Careful dissection is needed to expose the deep fascia over the pes anserinus and superficial fibers at the anterior margin of the medial collateral ligament (MCL). Once completing a sharp L-shaped incision along anterior border of the MCL and upper margin of the semitendinosus tendon, the MCL was raised and peeled off using a periosteal elevator. The first guide wire was then inserted toward a “safe zone,” with a starting point 3 mm below the upper margin of the semitendinosus tendon and anterior one-third of the medial tibial surface [17]. A second guide wire was also inserted 1 cm posterior to the first guide wire and advanced parallel to it under fluoroscopic guidance, so the osteotomy plane could mirror the posterior tibial slope. Finally, a Mini-Hohmann retractor was inserted beneath MCL, and multiple drill holes were made along the superior margins of both guide wires.

**Figure 1:**
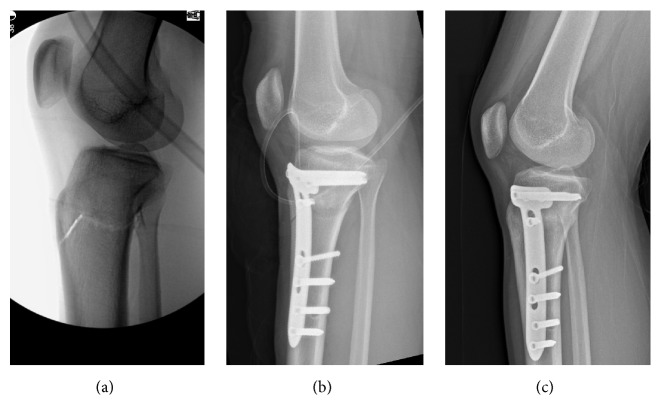
Lateral radiographs of open wedge high tibial osteotomy with distal tubercle osteotomy: (a) biplanar retrotubercle osteotomy (intraoperative view); (b) locking plate fixation at osteotomy site (immediately postoperative); and (c) union of the osteotomy site (postoperative month 3).

The first tibial osteotomy started at the center of the medial cortex and ran obliquely upward toward the fibular head to about 2 cm below the lateral joint line. The second osteotomy involved a complete transverse cut of the posterior cortex, extending from the posteromedial to posterolateral border. The last osteotomy of anterior cortex was directed proximal to the tibial tuberosity in group 1, using a biplanar approach in group 2 for technical reasons. The latter commenced at the anterior third of the main osteotomy, making a short beak distally; screw fixation was not required distally (beneath pes anserinus tendon), unless the beak fractured during osteotomy (Figure 1).

After completing the osteotomy, two 6-mm half-pins were inserted at the medial aspect of the proximal tibia for temporary fixation. The half-pin inserted proximally was placed 1–1.5 cm distal to the joint line and parallel to the posterior cortex of the tibial shaft. The distally inserted pin was placed just anterior to the posterior tibial cortex. A wedge-shaped spreader was then gently inserted into the osteotomy site and the gap was widened to correct the varus deformity. It is imperative to keep the knee fully extended during this maneuver to avoid unwanted changes in posterior tibial slope. Additionally, these half-pins can also be used to check on the changes in rotational alignment after varus correction. The spreader was locked when sufficient varus correction was evident grossly. A 15-cm carbon rod with three Schanz pin clamps was used to connect the two tibial half-pins.

**Figure 2:**
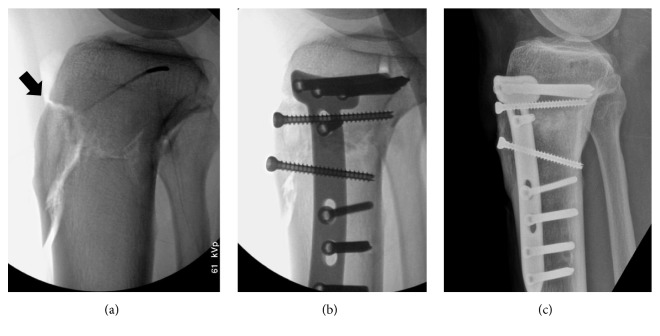
Images of patient with tibial tuberosity fracture: (a) fracture of proximal tibial tuberosity (arrow) in C-arm image; (b) fixation via two additional cannulated screws; and (c) healed fracture (postoperative month 3).

Keeping the fixator in place, gaps were filled with hydroxyapatite or ß-tricalcium phosphate (Bongros; CGBio, Seongnam-si, South Korea) or allograft cancellous bone chips (OSG-DualPor; OssGen, Gyungsan-si, South Korea). A plate (TomoFix®, TomoFix Medial High Tibial Plate; DePuy Synthes, Raynham, MA, USA) inserted into anteromedial tibial surface was then fixed in a less invasive manner, using 5.0-mm locking screws. In patients of group 2 (OWHTO/DTO) sustaining tibial tuberosity fractures, tibial tubercle was compressed against the distal tibia by anteroposterior countersinking of a 4.5-mm cortical lag screw (Figure 2). The wounds were irrigated and suction drains inserted.

Postoperative management was the same for both groups of patients. From postoperative day 1, gentle active range-of-motion knee regimens and full weight bearing were allowed as tolerated.

### 2.3. Radiographic Measures

All radiologic measurements were performed by two orthopedic residents who were blinded to the study. The measurements were independently generated, twice for each radiograph, with at least 1 month between assessments. To limit the number of variables, only the right tibia of each patient was analyzed.

Radiologic assessments were based on standing full-leg radiographs (patella pointing forward) and standing anteroposterior and lateral knee views obtained preoperatively and at the latest follow-up. Each standing lateral view was taken with the knee in 30-degree flexion and the limb in neutral position.

**Figure 3:**
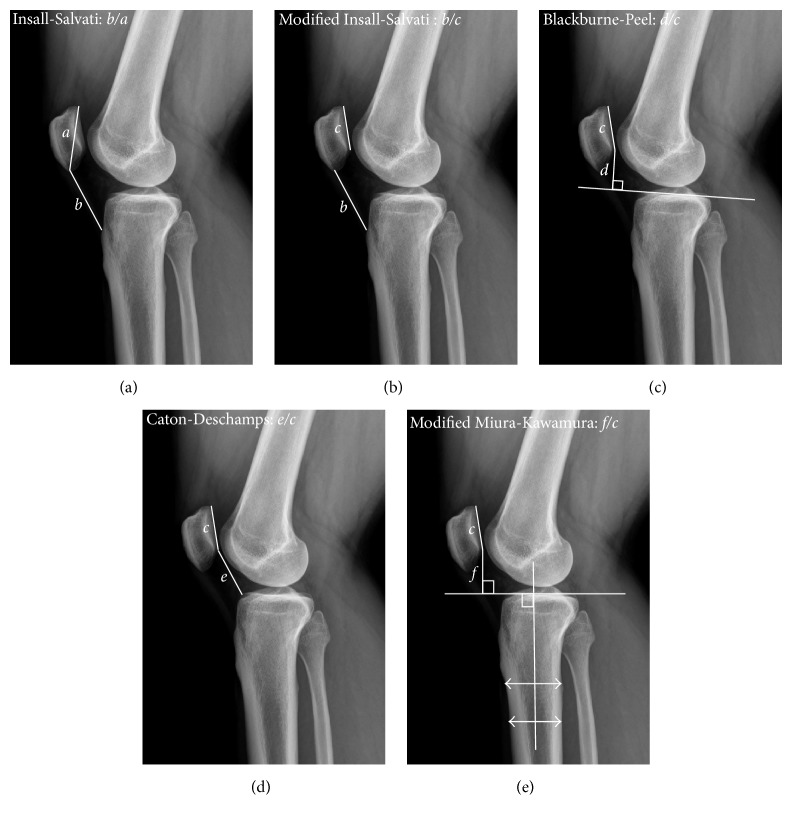
Radiographic diagrams showing five indices measured to assess patellar height: (a) Insall-Salvati; (b) modified Insall-Salvati; (c) Blackburne-Peel; (d) Caton-Deschamps; and (e) modified Miura-Kawamura. *a*, diagonal patellar length; *b*, patellar length; *c*, length of the patellar articular surface; *d*, perpendicular distance between the proximal tibial articular surface and the inferior tip of patellar articular surface; *e*, distance from the anterosuperior border of the tibial plateau to the inferior tip of the patellar articular surface; *f*, distance from a line perpendicular to the tibial diaphyseal line to the inferior tip of the patellar articular surface.

Coronal alignment was gauged via standing full-leg radiographs, measuring medial proximal tibial angle (MPTA), as described by Paley and Tetsworth [18], and mechanical femorotibial angle (mFTA) (positive and negative values indicating genu valgus and varum, resp.). The femoral mechanical axis passes from the center of the hip through the center of the knee, and the tibial mechanical axis passes from the center of the tibial spine through the center of the ankle mortise. The angle between these two axes corresponds to the mFTA. Patellar height was evaluated in standing lateral knee view by measuring the Insall-Salvati (IS) index [19], modified IS (mIS) index [20], Blackburne-Peel (BP) index [21], Caton-Deschamps (CD) index [22], and modified Miura-Kawamura (MK) index (Figure 3) [23]. The tibial slope was also calculated by the proximal tibial anatomic axis method, measuring the angle formed between the proximal medial plateau and a line drawn perpendicular to the tibial shaft axis.

**Figure 4:**
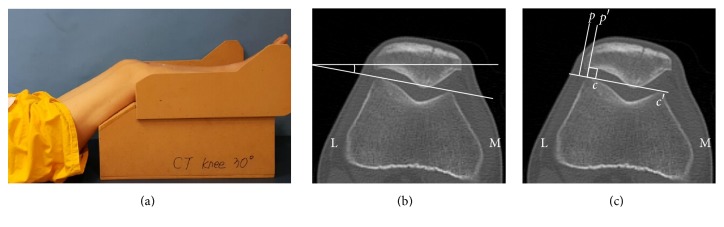
Methods of assessing lateral patellar tilt (LPT) and lateral patellar shift (LPS): (a) patient positioned for computed tomography imaging with knee in 30 degrees of flexion; (b) LPT, defined as the angle between a line intersecting the widest bony structure of the patella and a line tangential to the anterior femoral condylar surfaces; (c) LPS, defined as the ratio of distances *pp*′/*cc*′, where *cc*′ is the distance between the summits of the medial and lateral femoral condyles and *pp*′ is the distance between the summit of the lateral femoral condyle and the point where a line from the lateral edge of the patella perpendicular to the line that passed through the summits of the femoral condyles crosses that line. L, lateral; M, medial.

Lateral patellar tilt (LPT) and lateral patellar shift (LPS) were measured on axial images, generated pre- and postoperatively by two-dimensional CT [24–27]. CT scan slices were focused on the central patella, with hips flexed at 45 degrees and knees flexed at 30 degrees (Figure 4).

### 2.4. Statistical Analysis

All statistical calculations relied on standard software (SPSS v19.0, SPSS Inc., Chicago, IL, USA). All continuous variables were tested for normality via Shapiro-Wilk test and did not violate the normal distribution assumption. Student's paired *t*-test was used to assess differences between pre- and postoperative measures in each group. To compare changes in patellar position by operative group, Student's independent *t*-test was applied. Statistical significance was set at *p* < 0.05. Changes in patellar height and tibial slope and degrees of correction angle in the coronal plane (MPTA) were analyzed using Pearson's correlation coefficient. Inter- and intraobserver reliabilities were gauged as well through intraclass correlation coefficients (ICCs). ICCs were interpreted as follows: poor, <0.4; marginal, >0.4 but <0.75; and good, >0.75.

## 3. Results

Baseline demographics, as well as pre- and postoperative MPTA and mFTA data, are reported by operative method in Table 1. The two groups did not differ significantly in terms of age, gender, BMI, or follow-up period. There were no significant between-group differences with respect to mean preoperative MPTA and mFTA or mean extent of correction.

Pre- and postoperative patellar height indices, tibial slope, and lateral patellar tilt and shift are shown by group in Table 2. None of the pre- and postoperative variables differed significantly.

**Figure 5:**
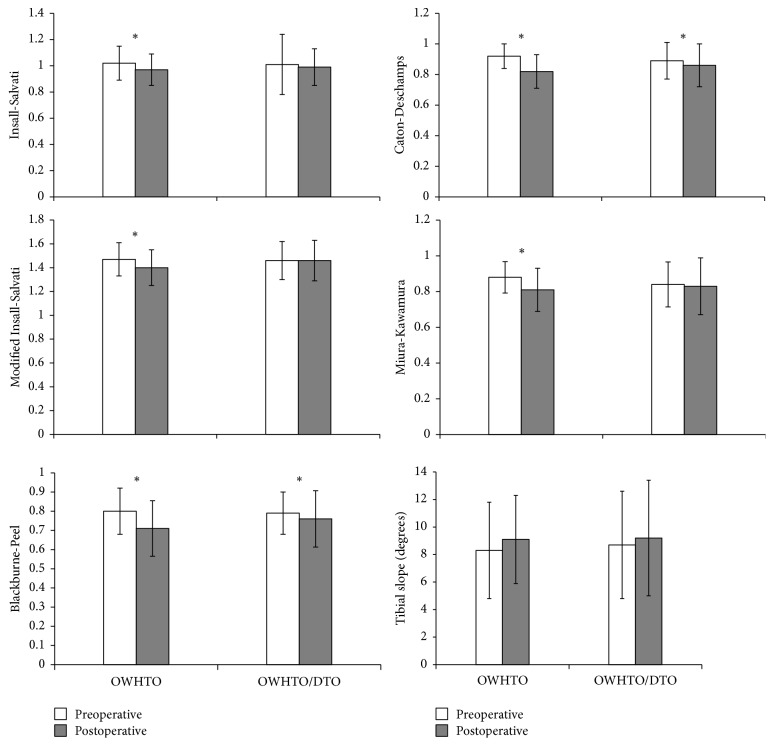
Patellar height indices and tibial slope pre- and postoperatively, charted by operative group. OWHTO, open wedge high tibial osteotomy; DTO; distal tubercle osteotomy. ^*∗*^Significant change after surgery (*p* < 0.05).

Patellar height and tibial slope measurements before and after surgery for both groups are tabulated in Figure 5. In group 1 (OWHTO), all patellar height indices including IS (*p* = 0.003), mIS (*p* = 0.007), BP (*p* < 0.001), CD (*p* < 0.001), and MK index (*p* < 0.001) declined significantly. Although mean BP (*p* = 0.023) and CD (*p* = 0.009) indices decreased significantly in group 2 (OWHTO/DTO), other patellar height indices were not significantly altered by surgery. Neither group showed significant change in mean tibial slope.

**Figure 6:**
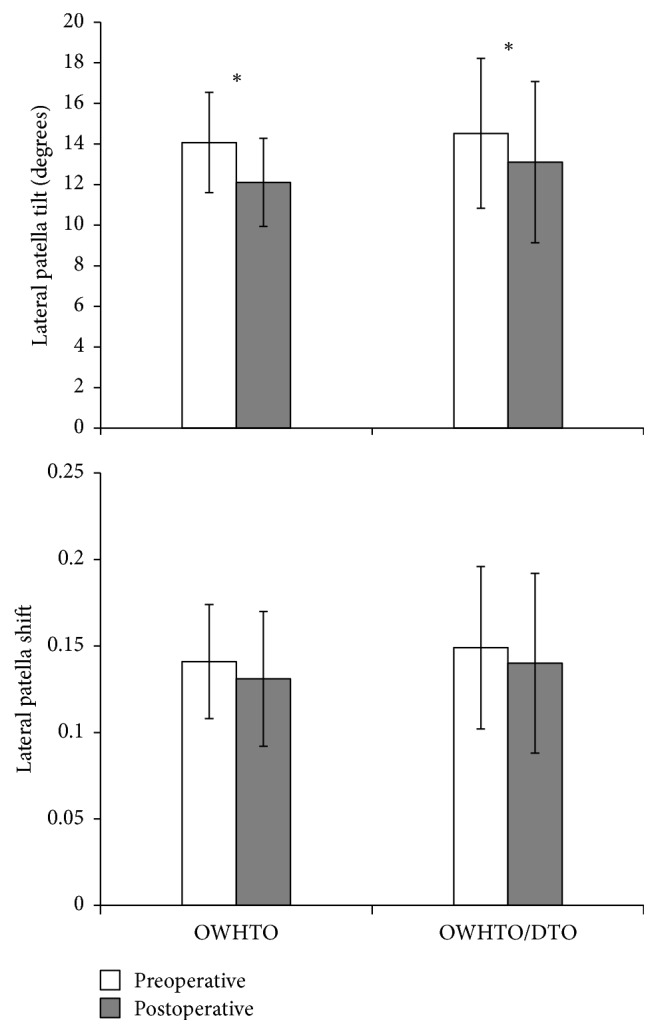
Lateral patellar tilt and lateral patellar shift pre- and postoperatively, charted by operative group. OWHTO, open wedge high tibial osteotomy; DTO, distal tubercle osteotomy. ^*∗*^Significant change after surgery (*p* < 0.05).

Average LPT decreased significantly after surgery in both groups. However, no statistical differences between preoperative and postoperative LPS were evident (Figure 6).

We compared the amount of change between group 1 and group 2 (Table 3). The amount of change of mIS, BP, CD, and MK indices significantly differed between two groups. Because of the small number of subjects, independent *t*-test power calculation was carried out using Power Analysis and Sample Size 11 for Windows software package (NCSS Inc., LLC, Kaysville, UT, USA), and the power was 0.76 for mIS index, 0.41 for BP index, 0.64 for CD index, and 0.73 for MK index between group 1 and group 2.

There were no significant correlations in either group with respect to patellar height or tibial slope changes or degree of correction angle in coronal plane (MPTA).

Bone union at osteotomy sites was achieved at an average of 3 months in all patients. A total of two complications occurred in group 2. In these two patients, the tibial tuberosity fractured during tuberosity osteotomy. Remedied by fixation screws, these fractures healed in 3 months. No other complications, such as wound infection, delayed union, or nonunion, were seen.

Interrater reliability of all patellar indices measured was confirmed using (ICCs) based on radiographs before and after surgery. ICC ranges for intraobserver (0.75–0.91) and interobserver (0.72–0.9) reliabilities were comparable to those of previous studies [14, 28].

## 4. Discussion

Introduction of OWHTO/DTO was fueled by efforts to minimize surgery-related loss in patellar height and prevent subsequent patellofemoral problems [8]. Several studies offer proof that this technique is effective in minimizing postoperative patellar height change, citing clinical outcomes comparable to conventional OWHTO in patients with osteoarthritis of the medial tibiofemoral compartment and varus malalignment [8, 14, 15, 29–31]. However, there was no study investigating the surgical results of OWHTO/DTO in patients with normal knee. Special concerns in this population include unsightly aesthetics and pain or discomfort upon kneeling after surgery. Therefore, we have used this technique to correct proximal tibial varus deformities in young patients without pain or arthritic joints, proving that diminished patellar height is avoidable through this approach. Considering the positional changes of the patella following conventional OWHTO may induce new symptoms, these concerns will be addressed by OWHTO/DTO.

In the present study, we found significant postoperative decreases in IS and mIS indices of the OWHTO group, whereas these ratios were unchanged in the OWHTO/DTO group. Outcomes herein were aligned with previous studies that similarly utilized these ratios to evaluate patellar height [14, 15, 29]. Both IS and mIS are indirect measures of patellar height, any change reflecting altered patellar tendon length [14, 32]. Any slackening or shortening of patellar tendon thus implied may be explained by intratendinous fibrosis or adhesions of proximal tuberosity following OWHTO, whereas the extensor mechanism remains intact after OWHTO/DTO.

Although there were significant postoperative declines in BP and CD indices of both groups, the MK index of the OWHTO/DTO group was unchanged. Still, only one other study corroborates our results [14]. Most earlier investigations have recorded no significant changes in BP and CD indices after OWHTO/DTO [8, 15, 29, 30]. This discrepancy may be due to the method of measurement. Both BP and CD indices have been viewed as highly reliable gauges of patellar height [28, 33], but their dependency on joint line is disadvantageous, given tibial plateau as a reference. Because tibial slope decreased postoperatively in both arms of our study, even minimal deviations in tibial slope may thus have bearing. We agree with other investigators that BP and CD indices should not be relied upon in surgical procedures involving proximal tibia [23, 32]. In our opinion, the decrease seen in MK index reflects a true reduction in patellar height because MK index is independent with the change of tibial slope.

Although various radiographic techniques (based on axial radiography, CT, or MRI) have been described for evaluating patellar inclination [34], use of LPT and LPS after OWHTO/DTO has not previously been investigated. Previous publications studying changes in LPT and LPS after OWHTO have relied on axial radiography of load-bearing patellofemoral joints (Delgado-Martins method) [7, 24, 35, 36]. However, determinations rendered by axial radiography may be not accurate due to overlapping of images, difficulty in positioning the leg, and incidental angles of X-ray beams [37, 38]. CT imaging of contracted quadriceps muscle may serve better in confirming patellar position [25, 34, 37, 39]. Consequently, we used axial CT images in supine position, with the knee in 30 degrees of flexion, to measure LPT and LPS.

Ultimately, we found that LPT declined significantly for both procedures, but the reductions did not differ significantly by operative group. Our findings following OWHTO were consistent with those of previous studies [35, 36], whereby lessening of LPT was attributed to increased lateral pull on the patella. Although we expected that OWHTO/DTO would prevent any changes in LPT and LPS, LPT surprisingly showed significant decline after OWHTO/DTO as well. One possible explanation for this is that a change in mechanical axis may affect the inclination of patella. For another reason, the change of tibial rotation after OWHTO may affect the LPT. Previous studies reported OWHTO made significant internal axial rotation of the tibia [40, 41]. However, there was no change on tibial rotation after varus correction because we used two half-pins to check the change of rotational alignment in the operating room.

In our analysis, no significant correlations emerged with respect to change in patellar height, change in tibial slope, or correction angle degree. Prior reports have indicated that OWHTO/DTO is more effective than conventional OWHTO in patients requiring large (>10-degree) angular corrections [8, 14, 15, 30]. However, we could provide corroboration, because correction angles for most of our patients were <10 degrees, and the surgical goal was to restore neutral alignment. Hence, the protective effect of OWHTO/DTO on patellar height after large angular correction (>10 degrees) could not be fully evaluated by this study.

There were two tibial tuberosity fractures in OWHTO/DTO group. Only one previous literature reported same surgical complications [8]. There was no such complication in other reports using OWHTO/DTO [14, 15, 29, 30]. Therefore, it seems that tibial tuberosity fracture is related to mistake of surgical procedure, not the degrees of correction angle. Additionally, in our study, such fractures occurred at an early stage in our learning curve and healed well with additional screw fixation. Eventually, we think that tibial tuberosity fracture is easily preventable with caution, leaving at least a 1-cm thickness on the proximal tuberosity fragment.

Our study has some limitations. First, it is a retrospective investigation and not a randomized trial. Also, the two operative groups reflected a shift in surgical technique rather than pure randomization. Nevertheless, the two groups were well matched at baseline in terms of preoperative variables. Another limitation was that blinding of the OWHTO/DTO arm was not feasible when measuring radiologic parameters postoperatively, even if two independent orthopedic residents participated. Finally, radiographic parameters were the primary focus. To investigate the relationship between radiologic findings and clinical outcomes, a well-planned future prospective study is needed.

There are various methods for correcting proximal tibial vara deformity, and best surgical technique must be individualized. Compared to conventional OWHTO, OWHTO/DTO did not influence patella height associated with the procedure and was considered an useful method to preserve patella height.

## Figures and Tables

**Table 1:** Demographics and correction angles by operative group.

Variables	OWHTO (*n* = 30)	OWHTO/DTO (*n* = 33)	*p* value
Age (years)	32.8 ± 7.5	30.5 ± 8.0	0.18
Gender (male : female)	8 : 22	13 : 20	0.23
Body mass index (kg/m^2^)	23.3 ± 5.2	24.5 ± 5.0	0.52
Preoperative MPTA (degrees)	81.8 ± 1.9	82.3 ± 2.3	0.30
Postoperative MPTA (degrees)	88.1 ± 2.0	88.3 ± 2.4	0.63
Correction of MPTA (degrees)	6.2 ± 2.1	6.0 ± 2.7	0.64
Preoperative mFTA (degrees)	−6.0 ± 1.8	−6.4 ± 3.0	0.44
Postoperative mFTA (degrees)	0.8 ± 1.5	0.3 ± 1.8	0.24
Correction of mFTA (degrees)	6.8 ± 2.3	7.7 ± 3.1	0.24
Follow-up period (months)	32.4 ± 8.7	33.1 ± 2.9	0.68

Values expressed as mean ± SD; OWHTO, open wedge high tibial osteotomy; DTO, distal tubercle osteotomy; MPTA, medial proximal tibial angle; mFTA, mechanical femorotibial angle.

**Table 2:** Pre- and postoperative radiographic measurements by operative group.

Variables	OWHTO (*n* = 30)	OWHTO/DTO (*n* = 33)	*p* value
Preoperative Insall-Salvati	1.02 ± 0.13	1.01 ± 0.23	0.932
Postoperative Insall-Salvati	0.97 ± 0.12	1.00 ± 0.14	0.353
Preoperative modified Insall-Salvati	1.48 ± 0.13	1.46 ± 0.16	0.701
Postoperative modified Insall-Salvati	1.40 ± 0.15	1.47 ± 0.17	0.072
Preoperative Blackburne-Peel	0.80 ± 0.11	0.79 ± 0.11	0.800
Postoperative Blackburne-Peel	0.71 ± 0.15	0.76 ± 0.15	0.138
Preoperative Caton-Deschamps	0.93 ± 0.08	0.90 ± 0.12	0.237
Postoperative Caton-Deschamps	0.83 ± 0.11	0.86 ± 0.14	0.265
Preoperative modified Miura-Kawamura	0.89 ± 0.08	0.84 ± 0.12	0.077
Postoperative modified Miura-Kawamura	0.81 ± 0.12	0.83 ± 0.16	0.552
Preoperative tibial slope (degrees)	8.3 ± 3.5	8.7 ± 3.9	0.655
Postoperative tibial slope (degrees)	11.1 ± 3.23	11.2 ± 4.16	0.943
Preoperative lateral patella tilt (degrees)	14.1 ± 2.47	14.5 ± 3.69	0.582
Postoperative lateral patella tilt (degrees)	12.1 ± 2.18	13.1 ± 3.98	0.249
Preoperative lateral patella shift	0.14 ± 0.03	0.15 ± 0.47	0.493
Postoperative lateral patella shift	0.13 ± 0.04	0.14 ± 0.05	0.450

Values expressed as mean ± SD; OWHTO, open wedge high tibial osteotomy; DTO, distal tubercle osteotomy.

**Table 3:** The amount of change for radiographic measurements by operative group.

Variables	OWHTO (*n* = 30)	OWHTO/DTO (*n* = 33)	*p* value
Insall-Salvati	−0.05 ± 0.08	−0.02 ± 0.20	0.408
Modified Insall-Salvati	−0.08 ± 0.14	0.00 ± 0.09	0.002
Blackburne-Peel	−0.09 ± 0.09	−0.04 ± 0.13	0.037
Caton-Deschamps	−0.10 ± 0.09	−0.04 ± 0.11	0.008
Modified Miura-Kawamura	−0.08 ± 0.09	−0.01 ± 0.12	0.010
Tibial slope (degrees)	0.78 ± 3.06	0.47 ± 3.95	0.705
Lateral patella tilt (degrees)	−2.06 ± 1.76	−1.41 ± 2.14	0.218
Lateral patella shift	−0.01 ± 0.03	−0.01 ± 0.03	0.661

Values expressed as mean ± SD; OWHTO, open wedge high tibial osteotomy; DTO, distal tubercle osteotomy.
